# Cross-modal decoding of emotional expressions in fMRI—Cross-session and cross-sample replication

**DOI:** 10.1162/imag_a_00289

**Published:** 2024-09-23

**Authors:** Lara A. Wallenwein, Stephanie N.L. Schmidt, Joachim Hass, Daniela Mier

**Affiliations:** Department of Psychology, University of Konstanz, Konstanz, Germany; Faculty of Applied Psychology, SRH University Heidelberg, Heidelberg, Germany

**Keywords:** mirror neuron system, machine learning, multivariate pattern analysis, social cognition, imitation, faces

## Abstract

The theory of embodied simulation suggests a common neuronal representation for action and perception in mirror neurons (MN) that allows an automatic understanding of another person’s mental state. Multivariate pattern analysis (MVPA) of functional magnetic resonance imaging (fMRI) data enables a joint investigation of the MN properties cross-modality and action specificity with high spatial sensitivity. In repeated-measures and independent samples, we measured BOLD-fMRI activation during a social-cognitive paradigm, which included the imitation, execution, and observation of a facial expression of fear or anger. Using support vector machines in a region of interest and a searchlight-based within-subject approach, we classified the emotional content first within modalities and subsequently across modalities. Of main interest were regions of the MN and the emotional face processing system. A two-step permutation scheme served to evaluate significance of classification accuracies. Additionally, we analyzed cross-session and cross-sample replicability. Classification of emotional content was significantly above chance within-modality in the execution and imitation condition with replication across sessions and across samples, but not in the observation condition. Cross-modal classification was possible when trained on the execution condition and tested on the imitation condition with cross-session replication. The searchlight analysis revealed additional areas exhibiting action specificity and cross-modality, mainly in the prefrontal cortex. We demonstrate replicability of brain regions with action specific and cross-modal representations of fear and anger for execution and imitation. Since we could not find a shared neural representation of emotions within the observation modality, our results only partially lend support to the embodied simulation theory. We conclude that activation in MN regions is less robust and less clearly distinguishable during observation than motor tasks.

## 1 Introduction

Mirror neurons (MN) in monkeys fire both when performing a movement and when observing a similar movement in others (Rizzolatti & Craighero, 2004). This mirroring mechanism has been proposed to allow the understanding of others’ actions, emotions, and mental states (Bonini et al., 2022;Gallese, 2007). However, the examination of MN in humans is often based on indirect measurements with limited sensitivity. Therefore, new analysis techniques such as multivariate pattern analysis (MVPA) classification are required to assess fine-grained neural activation patterns.

Since the discovery of MN in the premotor brain area F5 of macaque monkeys (di Pellegrino et al., 1992), this common neural representation of observed and performed actions has been subject to a great body of research (e.g.,Geiger et al., 2019;Iacoboni et al., 2005;Kohler et al., 2002;Oberman et al., 2005). The embodied simulation theory (e.g.,Gallese, 2007;Gallese & Goldman, 1998) assigns a functional role to the MN system in human social cognition: Through embodiment of actions we observe in others, we gain an automatic understanding of this person’s mental state, goals, and intentions. Different brain regions have been proposed to be part of the human MN system. Besides regions directly involved in performing motor actions (such as the ventral premotor cortex), also the inferior frontal gyrus (IFG), the inferior parietal lobule (IPL), and the superior temporal sulcus (STS) have been proposed to constitute the human MN system (e.g.,Keysers & Gazzola, 2006). Meta-analyses confirmed the involvement of these regions in both observing and performing simple motor tasks (Bekkali et al., 2021;Molenberghs et al., 2012) and reported further activation in a variety of brain regions depending on the specific task (Molenberghs et al., 2012). For social-cognitive tasks, the meta-analysis byMolenberghs and colleagues (2012)additionally identified increased activation in the amygdala, insula, and cingulate gyrus for the execution and observation of emotional expressions. The involvement of the MN regions in concert with regions for emotion processing in social-cognitive processes has further been demonstrated in tasks assessing emotion recognition, empathy, and theory of mind (e.g.,Mier, Sauer, et al., 2010;Sadeghi et al., 2022;Schmidt et al., 2021). These regions have been argued to provide the shared neural basis for social cognition, as activation was found in the same voxels for all these social-cognitive tasks within the same persons (Schmidt et al., 2021).

Despite the considerable amount of research and positive findings, the nature of involvement of MN in human social cognition is still a matter of debate (Cook et al., 2014;Heyes & Catmur, 2022;Jacob & Jeannerod, 2005). Single cell recordings, as have been performed in monkeys, are mostly not feasible in humans due to ethical considerations. Therefore, non-invasive but more indirect approaches have been utilized in humans to assess whether regions exhibit the key MN properties cross-modality and action specificity (Oosterhof et al., 2013). It should be noted that in this context the term modality refers to the modality of an action (e.g., execution, observation, imagination, or imitation) while the action itself can involve movements from all body parts, in the case of the present project faces displaying distinct emotions. Numerous studies utilized regional convergence in mass-univariate functional magnetic resonance imaging (fMRI) analyses. Although these approaches can provide information about regional cross-modal responsiveness, they have been criticized for neglecting the second key property action specificity: It has been argued that cross-modal responsiveness within a voxel might stem from neighboring but distinctive neuronal populations coding for different actions or reflecting cognitive processes, such as attention, instead of the actions (for a detailed discussion, seeDinstein, Thomas, et al., 2008;Oosterhof et al., 2013). Another method used to assess MN are fMRI adaptation approaches with the rational that suppression of neuronal activation should not only occur when an action is repeated in the same modality, but also when repeated in another modality (Fuelscher et al., 2019). Although adaptation approaches can thus assess both requirements simultaneously, they rely on inhibitory neurophysiological mechanisms that are not fully understood (Grill-Spector et al., 2006) and results have been highly heterogeneous (Chong et al., 2008;de la Rosa et al., 2016;Fuelscher et al., 2019;Kilner et al., 2009;Lingnau et al., 2009;Schmidt et al., 2020). Cross-modal MVPA has been proposed as a method to assess both key properties in concert with high spatial sensitivity (Fuelscher et al., 2019;Oosterhof et al., 2013;Peelen & Downing, 2007). The within-subject classification further allows the assessment of individual activation patterns in contrast to group-based univariate analysis approaches. Additionally, it provides a more direct measure than adaptation approaches, as it does not rely on the neurophysiological adaptation processes. In cross-modal MVPA, a pattern classifier is trained to distinguish different types of movement in one modality and its ability to correctly classify the same movements in the other modality is assessed. If the same patterns can differentiate the movements in both modalities, the region demonstrates cross-modal and action specific representations.

A small number of studies adopted a cross-modal classification approach to investigate MN system activity in humans with fMRI. Regions demonstrating MN properties included the occipitotemporal cortex, anterior parietal cortex (Oosterhof et al., 2010,2012a,2012b), and premotor cortex (Etzel et al., 2008;Oosterhof et al., 2012a). However, in one of these studies, none of the regions of interest (ROI), including the premotor cortex and intraparietal sulcus, fulfilled the criteria for MN properties (Dinstein, Gardner, et al., 2008). It should be noted that the studies differed in classification approaches, assessed ROIs, examined sensory modalities, and some applied classification with limited sample sizes. In addition, these reported MVPA studies did not assess the involvement of MN in social cognition directly, but rather the representation of specific bodily movements, instead of facial expressions that form the primary basis of social information in daily interactions.

General neural representations of emotional face processing have been studied intensively. Brain regions identified to be crucially involved in emotion recognition are the amygdala, the STS, and the fusiform gyrus (FFG;Allison et al., 2000;Haxby et al., 2000). In this field, MVPA approaches have recently been utilized to assess distinct neural patterns in the representation of emotional expressions (Kragel & LaBar, 2014). Regions with classification accuracy of different facial expressions above chance included the STS, amygdala, and FFG, among others (Greening et al., 2018;Harry et al., 2013;Said et al., 2010;Wegrzyn et al., 2015;H. Zhang et al., 2016). However, in these studies, facial expressions were only observed and the MN property of cross-modality (i.e., shared representation of executed and observed facial expressions) has not been assessed. To our knowledge, only one study investigated the embodied simulation theory with cross-modal decoding of emotions.Volynets et al. (2020)found that neural signatures of joy, anger, and disgust were significantly decodable across modalities (i.e., observing and displaying emotional facial expressions) in somatomotor, face perception, and several emotion circuit regions, but not in amygdala.

In the present study, we examined the processing of facial expressions with a task designed to investigate the involvement of MN in social-cognitive processes. First, we classified the facial expressions of fear and anger separately within modalities, namely execution, observation, and imitation. Subsequently, we examined whether decoding of the facial expressions of fear and anger is possible across these modalities. Based on the present literature, we expected cross-modal neural representations in regions of the human MN system (i.e., IFG, IPL, posterior STS (pSTS)) and regions of the emotional face processing system (EFP; i.e., amygdala and FFG). To assess decodability without*a priori*regional confinement, we further adopted a whole-brain information mapping (“searchlight”) approach (Kriegeskorte et al., 2006), in addition to the ROI-based classifications. Currently, fMRI-within-subject-classification studies that assess reliability or apply a replication approach are also sparse (Han et al., 2022;Taxali et al., 2021). We had data from two studies: One included repeated scanning timepoints and the other a similar imitation paradigm. This allowed us to assess the replication of classification findings over different time points for the same participants, that is, cross-session replicability, and the replicability over different samples, that is, cross-sample replicability. To the best of our knowledge, thus far, only the study byVolynets et al. (2020)adopted a similar cross-modal MVPA approach to assess MN properties in a social-cognitive task. Additionally, we investigated both predefined ROIs and a searchlight analysis and assessed replicability of our findings.

## 2 Material and Methods

Data of two different studies are analyzed jointly in this project. In the first study, brain activation was assessed during three different social cognitive tasks of which only the imitation task will be investigated here (see alsoSchmidt et al., 2021). Participants underwent two acquisition timepoints. During the first session (S1.1), a simultaneous EEG-fMRI acquisition was conducted. Data of the imitation task of all participants of the first scanning session will be analyzed here. The second session (S1.2) involved transcranial magnet stimulation or sham stimulation prior to scanning. Only the participants receiving sham stimulation are of interest for the present analyses. The second study (S2) assessed a similar imitation paradigm and had only one scanning session without simultaneous EEG or TMS, as well as two other tasks on the MN system, one of them applying an fMRI adaptation approach (Schmidt et al., 2020). Both studies were approved by the local ethics board of the Medical Faculty Mannheim, University of Heidelberg.

### 2.1 Participants

Participants for both studies were recruited via flyer and social media. Inclusion criteria were MRI-compatibility, no history of a neurological or mental disorders, and university entrance certification. All participants were informed about the procedure, aims, and potential risks of the study and signed written informed consent. The sample size was determined before data acquisition and was based on previous analyses on imaging genetics, including possible dropouts (Mier, Kirsch, et al., 2010). Eighty individuals were recruited for the first study and 81 for the second study. Of the total of 161, participants had to be excluded due to anatomical or further medical aberrations (4), technical issues (11), psychopathological aberrations despite exclusion of a history of mental disorders (4), multiple times extensive head movement of more than 3 mm translation or 3° rotation (2) or withdrawal from participation (3). Thus, data of 73 participants could be included for S1.1 (42 female, age*M = *23.51,*SD = *3.81), 32 for S1.2 (20 female, age*M = *23.97,*SD = *3.98), and 64 for S2 (38 female, age*M = *22.92,*SD = *3.4). In study 1, all participants reported to be university students except for one who did not disclose any information on their study or work status. In study 2, five participants had already finished their university studies, with one being a psychology PhD student, two being PhD students or researchers in a different or unknown subject, and two working in non-academic positions.

### 2.2 Data acquisition

Data were acquired at the Central Institute of Mental Health in Mannheim, Germany, using a 3T Siemens Magnetom Trio and a 12-channel head coil. For study 1, a Magnetization Prepared - RApid Gradient Echo (MPRAGE) was acquired with the following parameters: time of repetition (TR) = 1570 ms, echo time (TE) = 2.75 ms, flip angle = 15°, field of view (FOV) = 256 mm, matrix size = 256 × 256 mm, and voxel size of 1.0 x 1.0 x 1.0 mm^3^. During task performance, functional echo-planar images (EPI) were acquired with 32 descending slices, TR = 2000 ms, TE = 30 ms, flip angle = 80°, FOV = 192 mm, band width = 2112 Hz/Px, matrix size 64 x 64, slice thickness = 3 mm, and voxel size of 3.0 x 3.0 x 3.0 mm^3^. The MPRAGE sequence for study 2 was acquired with TR = 2300 ms, TE = 3.03 ms, flip angle = 9°, FOV = 192 mm, and a voxel size of 1.0 x 1.0 x 1.0 mm^3^. The EPI sequence was acquired with the same parameters as study 1 except for 33 slices and TE = 28 ms.

### 2.3 Paradigm

**Fig. 1.**
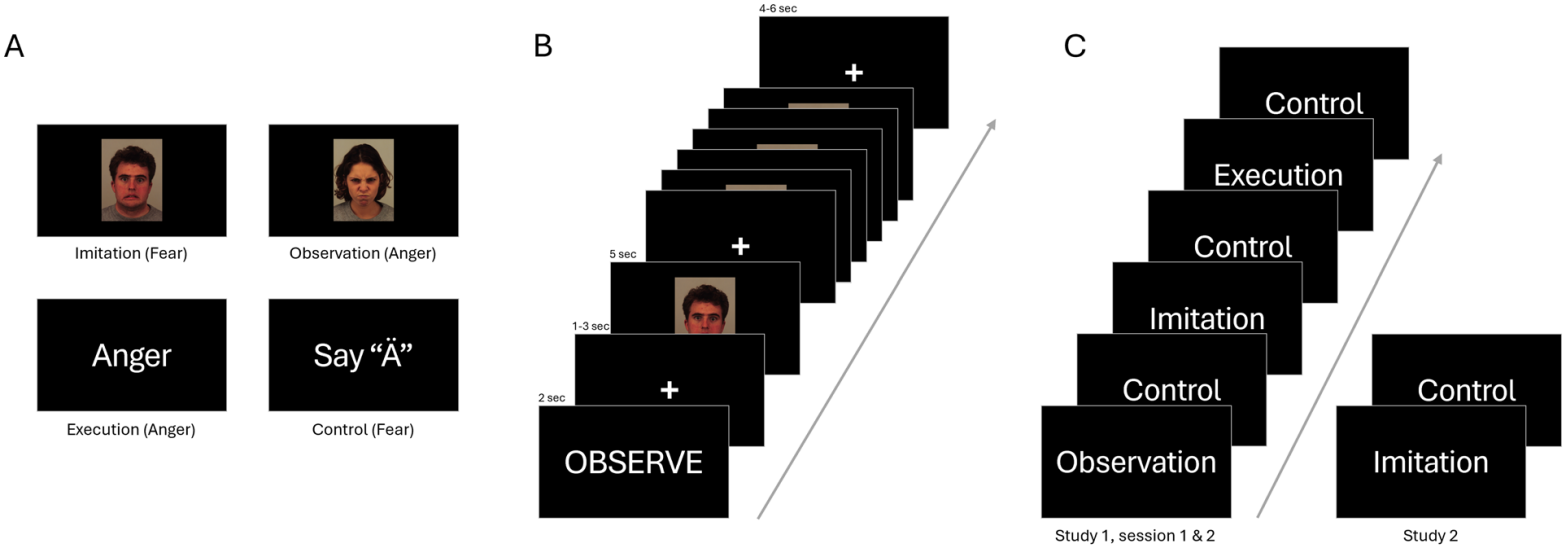
Task Paradigm. (A) Experimental conditions with exemplary stimuli. Pictures of angry and fearful facial expressions were shown in the imitation and observation condition. The words “anger” and “fear” were used in the execution condition and the letters “Ä” and “A” in the control condition. In the imitation and execution condition, participants performed the according emotion. In the control condition, participants pronounced the letter, and in the observation condition, the face was observed without an action. (B) Exemplary block of the observation condition with presentation durations. Each block consisted of two anger and two fear stimuli, except for the control condition in study 1, in which only 2 stimuli were shown per block. (C) Schematic task paradigms. Study 1 included all task conditions in each session. Study 2 included only the imitation and control conditions. In study 1, session 1 and study 2 stimuli from the Karolinska Directed Emotional Faces stimulus set were presented while for study 1 session 2 the NimStim Set of Facial Expressions was used.

The imitation paradigm performed during the task fMRI sequence and the univariate activation results are also described in detail elsewhere (Schmidt et al., 2021). Briefly, the imitation task employed in study 1 consisted of an imitation, an observation, an execution, and a control condition. For S1.1 and S2, facial stimuli from the Karolinska Directed Emotional Faces stimulus set (Lundqvist et al., 1998), depicting either an angry or a fearful emotional expression, were used in the imitation and the observation condition. For S1.2, stimuli from the NimStim Set of Facial Expressions (Tottenham et al., 2009) were chosen to avoid repetition effects in study 1. In the imitation condition, participants had to imitate the facial expression as precisely as possible while in the observation condition they were instructed to passively view the expression presented. In the execution and control condition, a cue indicated the facial expression participants were to perform. In the execution condition, participants should either perform an angry (cue word “Ärger”) or a fearful facial expression (cue word “Angst”) similar to the imitation condition though without viewing a facial stimulus. In the control condition, participants were presented with the cues “Ä” or “A” and were instructed to pronounce these German letters, resembling the expression of anger or fear, respectively (Fig. 1). In S1.1 and S1.2, each participant performed five blocks per experimental condition with four stimuli each and three control conditions in-between with two stimuli each. Thus, in total each experimental condition consisted of 20 stimuli (10 fearful, 10 angry) and the control condition of 30 stimuli. The order of blocks was fixed, emotion and gender were pseudorandomized within conditions. Each block of the imitation and observation condition had an equal number of male and female, as well as fear and anger stimuli. Duration of stimulus presentation was 5 sec in the experimental conditions and 3 sec in the control condition. Stimuli were separated by an inter-stimulus interval of 1–3 sec. Instruction cues were presented for 2 sec and were preceded by an inter-trial-interval of 4–6 sec. Total task duration was 13 min.

For S2, the paradigm consisted only of an imitation and a control condition. The imitation and control condition had the same procedure and timing as the experimental conditions described above with five blocks of the imitation and control conditions, each block consisting of 4 stimuli per condition. Each condition thus involved 20 stimuli (10 fearful, 10 angry), which were presented for 5 sec, respectively. Total task duration was 6 min.

### 2.4 Image data preprocessing and beta extraction

Image preprocessing and univariate first-level analyses were performed using SPM12 (https://www.fil.ion.ucl.ac.uk/spm/software/spm12) in MATLAB v2021a (The MathWorks, Inc., Natick, Massachusetts, USA). The fMRI images were slice time corrected, realigned to the mean fMRI image to correct for head motion, and unwarped. Thereafter, they were coregistered to the corresponding T1-weighted image and aligned to MNI space. No smoothing was applied. Visual quality checks were performed to ensure accurate coregistration and normalization. If movement parameters exceeded 3 mm or 3° only in peaks of maximum four consecutive volumes and no more than three times per participants, volumes were interpolated, and preprocessing was repeated. Interpolation was performed for four participants in S1.1, two participants in S1.2, and none in S2.

A general linear model was applied on the subject level. Each trial of each condition (i.e., imitation, observation, execution, and control for study 1, and imitation and control for study 2) was modeled in a separate regressor along with six motion parameters as covariates of no interest. Thus, we obtained parameter estimates (beta images) per trial that served as data input for subsequent MVPA analyses (e.g.,Mumford et al., 2012).

### 2.5 Multivariate pattern analyses

For ROI analyses, masks of BA44, IPL, amygdala, and FFG were retrieved from the WFU Pickatlas. As the atlas does not provide a parcellation for the pSTS, this mask was based on functional data of a previous study on social cognition (Mier, Lis, et al., 2010). Since we were interested in representations within systems, the single ROI masks were combined into masks for the MN system (IPL, IFG, and pSTS; size: 9758 voxels) and the EFP system (amygdala and FFG; size: 3827 voxels), respectively. The combined ROIs are depicted inFigures 2and5. The MN system is shown in blue and the EFP system in orange.

**Fig. 2.**
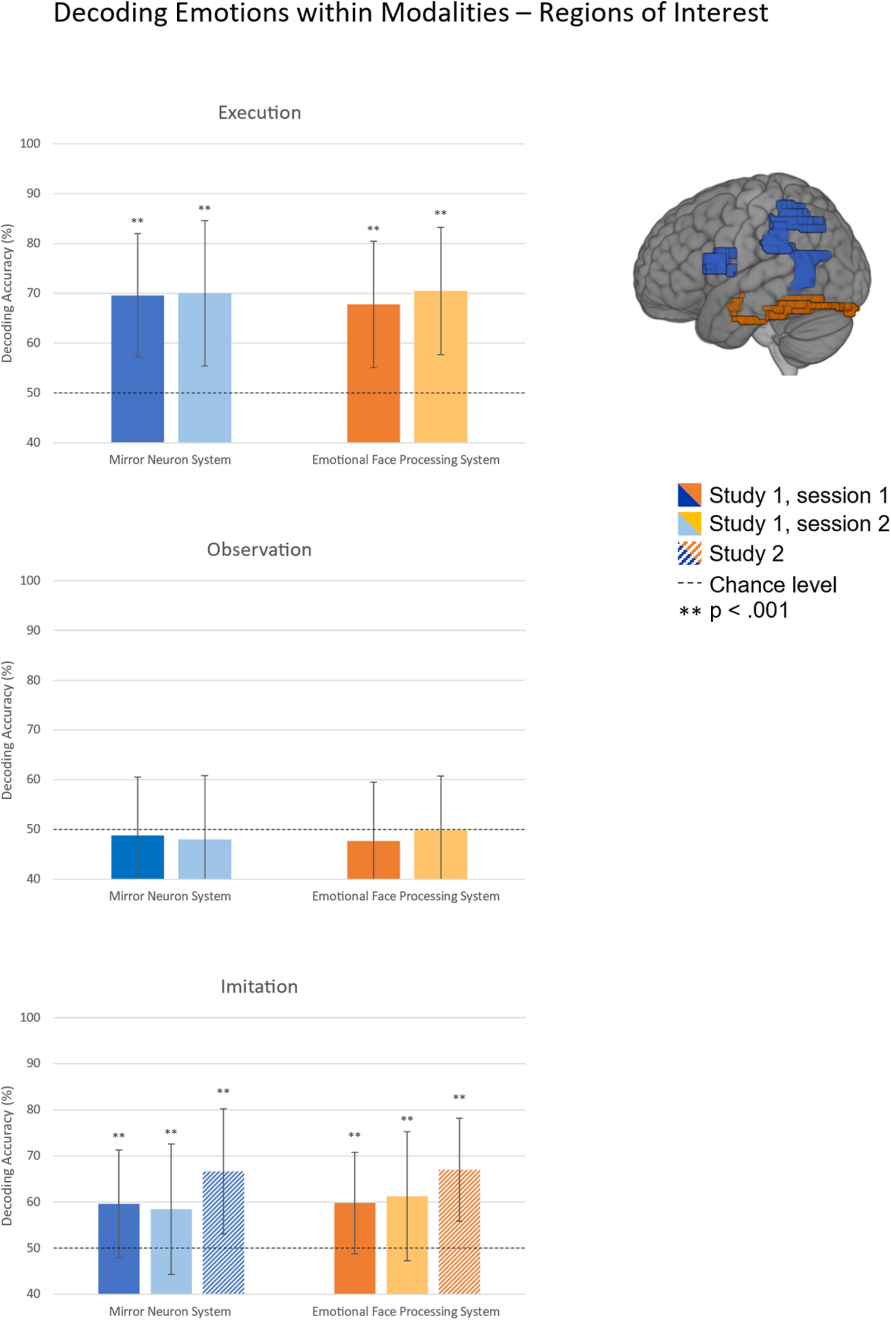
Decoding accuracy of emotional content (i.e., fear or anger) of regions of interest within the mirror neuron system and the emotional face processing system within modalities. Error bars represent standard deviations, significance above chance level (50%) evaluated based on the two-step permutation scheme.

For searchlight analyses, a radius of 3 voxels was chosen per searchlight (e.g.,Vermeylen et al., 2020). Thus, each searchlight contained 93 voxels. For each voxel in the brain, a sphere including neighboring voxels was constructed. MVPA was then performed for data from voxels within the sphere and the resulting decoding accuracy was assigned to the middle voxel. This results in a decoding accuracy map per participant per analysis (Kriegeskorte et al., 2006).

MVPA were performed using The Decoding Toolbox v 3.999 (TDT;Hebart et al., 2014) in MATLAB v2022a with default linear L2-norm support vector machines (SVM) as classifier and a fixed regularization parameter*C*= 1 implemented in LIBSVM (Chang & Lin, 2011). A classifier was trained separately for each participant per analysis. The resulting classification accuracies then entered a group level analysis to assess significance above chance level (50%).

To assess whether modalities are differentiable, we performed pairwise classification (i.e., observation and execution, imitation and observation, imitation and execution, and imitation and control), including all trials regardless of emotional content per modality. We used a 5-fold cross-validation (CV) “leave-one-block-out” scheme. Hence, in each fold, a classifier was trained on all trials of four task blocks and tested on trials of the left-out fifth block. Classification accuracies were averaged across the 5 CV folds per participant. The same significance testing and assessment of replicability were applied as described below. Results are reported inSupplementary Materials.

For hypothesis testing, we first classified the emotional content of the facial expressions (i.e., fear or anger) within the imitation, execution, and observation condition, respectively. The same 5-fold CV described above was applied. These analyses allowed us to assess whether fear and anger are differentiable and thus whether neural representations are action specific within the single modalities. Cross-sample replicability was assessed for the imitation condition by performing the same analysis in data of study 2.

Second, to assess both MN properties cross-modality and action specificity jointly, we applied cross-modal classification of the emotional content. For the training sets only data of the observation and execution conditions were used with the two other conditions (including imitation) as test sets, respectively. Imitation was not used for training with the reasoning that it should constitute a compound process of execution and observation (Schmidt et al., 2021). We thus performed training only on the subprocesses. For example, a classifier was trained to differentiate fear and anger in the execution condition and tested on data of the imitation condition. No CV scheme was required for these analyses, as training and test set were clearly distinct. All analyses within and across modalities were repeated in data of S1.2 to evaluate cross-session replication (i.e., training and testing were performed on data of S1.2). As study 2 only entailed the imitation condition as experimental condition, cross-sample replicability could not be assessed for the cross-modal analyses.

### 2.6 Significance testing

For significance testing, we applied a two-step permutation scheme based on the approach byStelzer et al. (2013)with permutations on the individual and group level. This non-parametric approach allows statistical inference on a group level based on within-subject classification results. First, 100 random label permutations were performed on the subject-level for each analysis. The structure of the original analyses (e.g., CV folds) was retained, respectively. Second, to assess significance on a group level, 10^5^random draws (with replacement) were computed. Per draw, one of the 100 permutation accuracies was chosen randomly per subject with a bootstrap (Monte Carlo) method. Subsequently, the mean permutation accuracy was computed within draw over all participants, resulting in 10^5^group permutation mean values. The original mean accuracy was tested against the distribution of these permutation means (right-tailed, α = .05), providing a significance estimate. This procedure was equivalently applied to ROIs and voxels in the searchlight analyses. Furthermore, a false-discovery rate (FDR)-correction with*α*= .05 was applied for searchlight analyses to correct for multiple comparisons. Cross-session replication was defined by voxels with a mean classification accuracy ≥ 60% in both sessions of study 1. Hence, if a region achieved this accuracy threshold in both sessions, we interpreted the classification ability as being replicable across sessions. Accordingly, the overlap of accuracies ≥ 60% in the same voxels in study 1 session 1 and study 2 provided a measure for cross-sample replicability.

## 3 Results

### 3.1 Decoding emotions within modalities

**Fig. 3.**
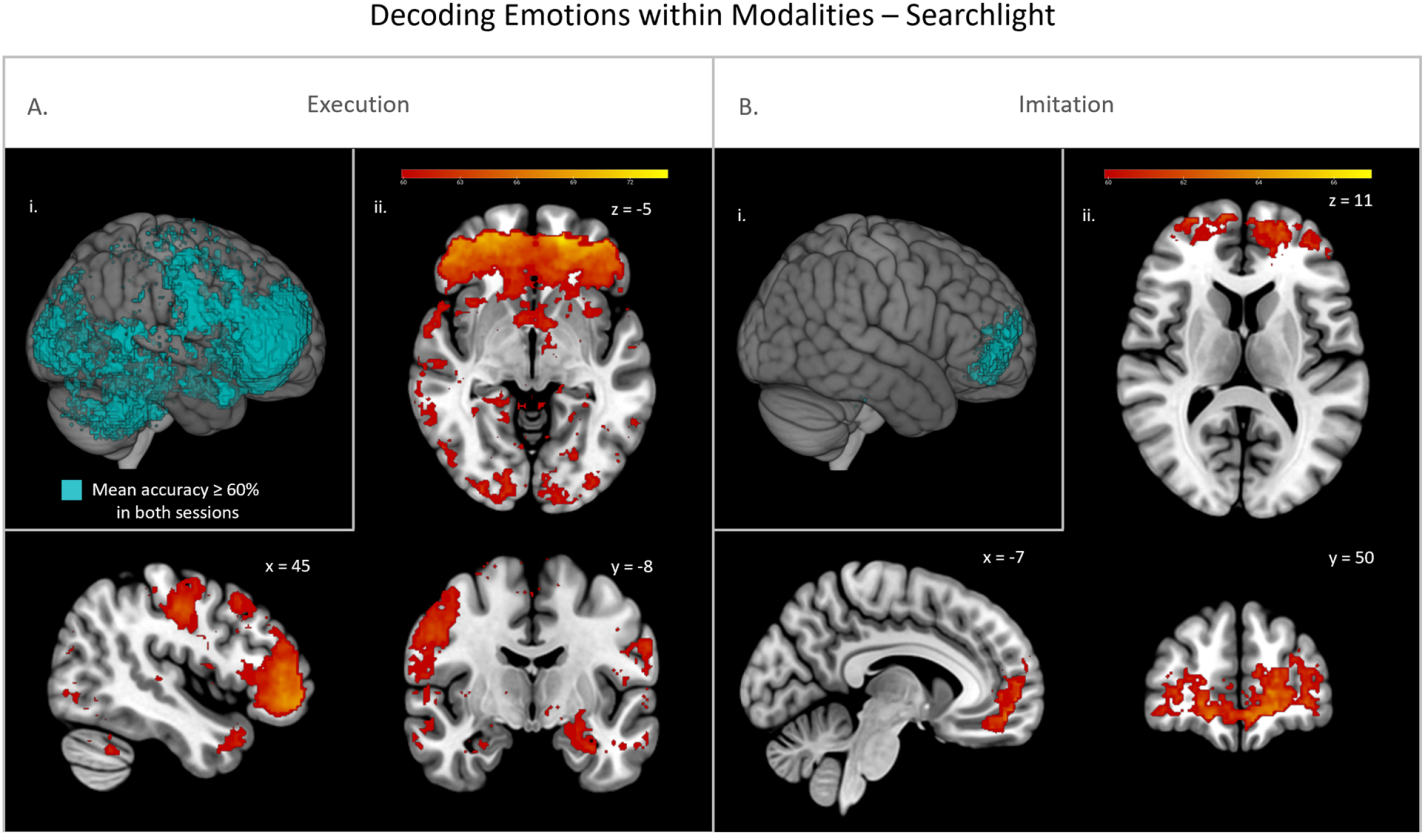
Searchlight analyses for the classification of emotions within the execution (A) and imitation condition (B) with cross-session replication. (i) Cross-session replication with overlapping significant voxels of study 1 session 1 (S1.1) and session 2 (S1.2) with mean accuracy ≥ 60% binarized. (ii) Mean classification accuracy of significant voxels (cut-off: mean accuracy ≥ 60%) of study 1 session 1 (S1.1) only.

**Fig. 4.**
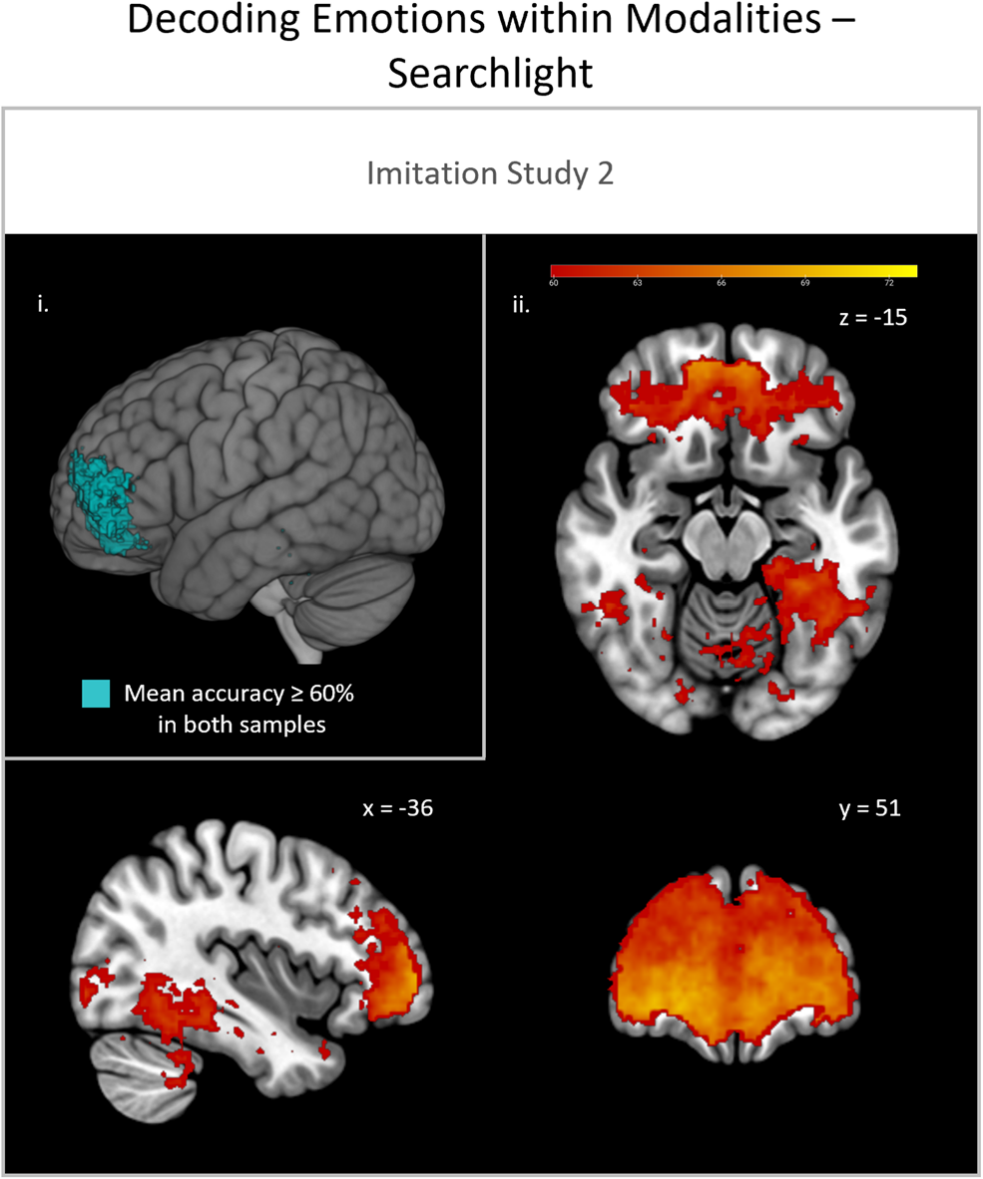
Searchlight analyses for the classification of emotions within the imitation condition with cross-sample replication. (i) Cross-sample replication with overlapping significant voxels of study 1 session 1 (S1.1) and study 2 (S2) with mean accuracy ≥ 60% binarized. (ii) Mean classification accuracy of significant voxels (cut-off: mean accuracy ≥ 60%) of study 2 (S2) only.

For the classification of emotional content within modalities (i.e., experimental conditions), we observed significant above chance classification accuracies in ROI analyses in both the MN system and the EFP system within the imitation and execution condition for all studies and sessions, respectively, but not within the observation condition. Mean accuracies of all samples are depicted inFigure 2and provided inTable 1along with mean area under the receiver operating characteristic curve (AUC) values. For the searchlight analysis within the execution condition, we observed a replicable effect across sessions in the prefrontal cortex, motor cortex, occipital cortex, cerebellum, and smaller clusters in more widespread areas (Fig. 3A). Within the imitation condition, we similarly observed a replicable effect in the prefrontal cortex with replication across sessions (Fig. 3B) and across samples (Fig. 4). Within the observation condition, no area with at least 60% mean accuracy in any of the sessions could be found.

**Table 1.** Classifier performance metrics for the classifications of emotional content in ROI analyses.

	Mirror neuron system						Emotional face processing system					
	Accuracy			AUC			Accuracy			AUC		
	S1.1	S1.2	S2	S1.1	S1.2	S2	S1.1	S1.2	S2	S1.1	S1.2	S2
Execution	69.59	70		75.18	76.2		67.74	70.47		73.45	77	
Observation	48.77	47.97		47.41	46.31		47.67	49.84		46.38	50.56	
Imitation	59.59	58.44	66.64	62.82	61.97	71.13	59.79	61.25	67.03	62.95	64.38	72.17
Training: execution, test: imitation	63.22	65.16		69.78	71.09		62.88	61.09		70.84	66.84	
Training: execution, test: observation	49.66	51.41		50.22	49.53		49.38	48.28		50.22	47.47	
Training: observation, test: imitation	51.03	51.56		49.51	53.66		48.7	53.44		49.62	54.66	
Training: observation, test: execution	50.75	52.5		50.84	51.88		53.56	50.94		51.92	52.16	

*Note.*Classification of fear and anger within Regions of Interest. Mean accuracy and area under the ROC curve per sample and analysis. AUC = area under the ROC curve, S1.1 = Study 1 session 1; S1.2 = Study 1 session 2; S2 = Study 2.

### 3.2 Decoding emotions across modalities

**Fig. 5.**
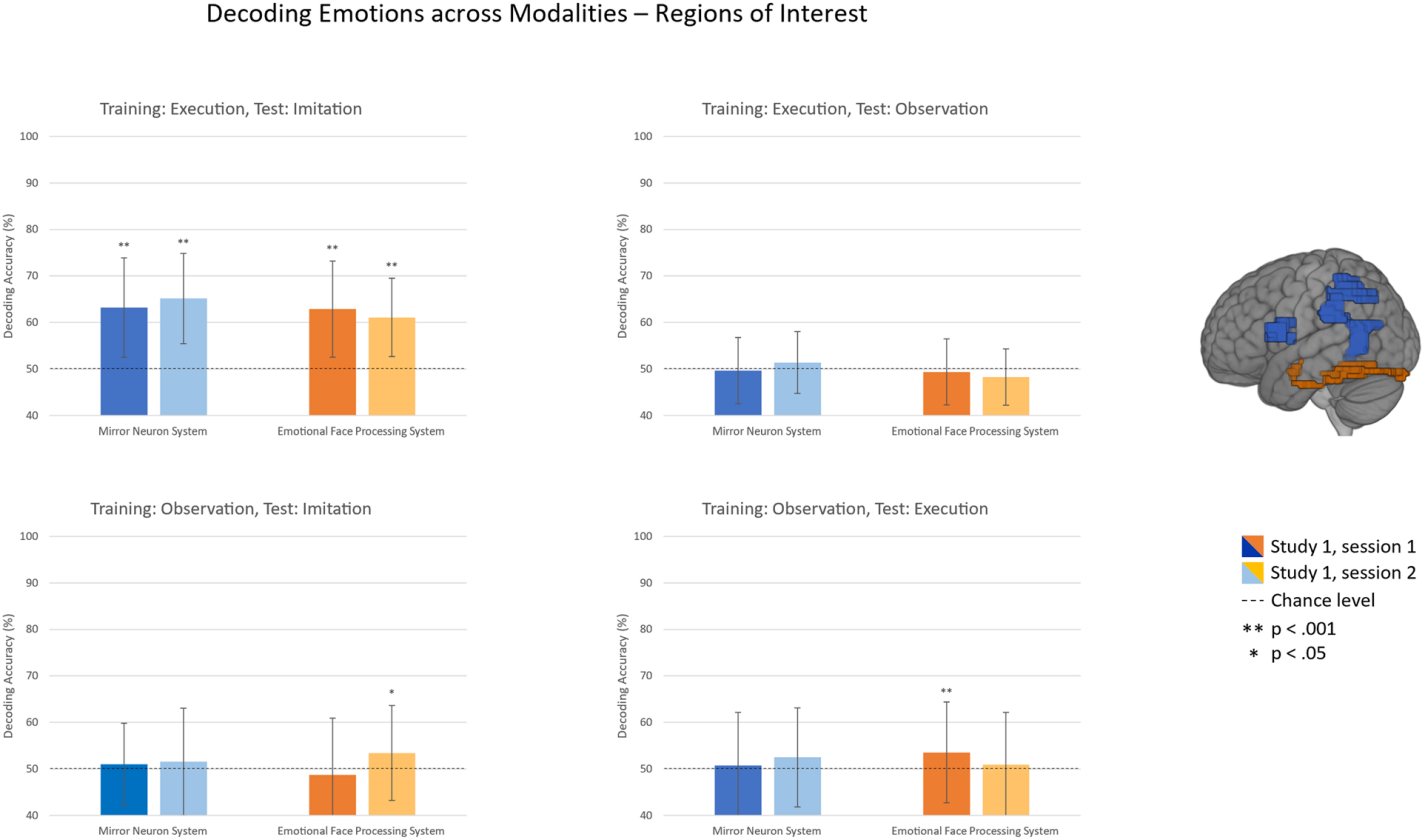
Decoding accuracy of emotional content (i.e., fear or anger) of regions of interest within the mirror neuron system and the emotional face processing system across modalities. Error bars represent standard deviations, significance above chance level (50%) evaluated based on two-step permutation scheme.

Cross-modal classification within ROIs was significant above chance with replication across sessions, when trained on the execution condition and tested on the imitation condition. When trained on the observation condition, cross-modal classification within the EFP system was found for imitation in the second session, but not the first session (S1.1), and for the execution condition in the first session, but not the second session (S1.2). Thus, cross-modal classification was not replicable, and we did not find any significant results for the MN system for these analyses. Furthermore, classification accuracies were not above chance level when trained on the execution condition and tested on the observation condition (Fig. 5andTable 1).

**Fig. 6.**
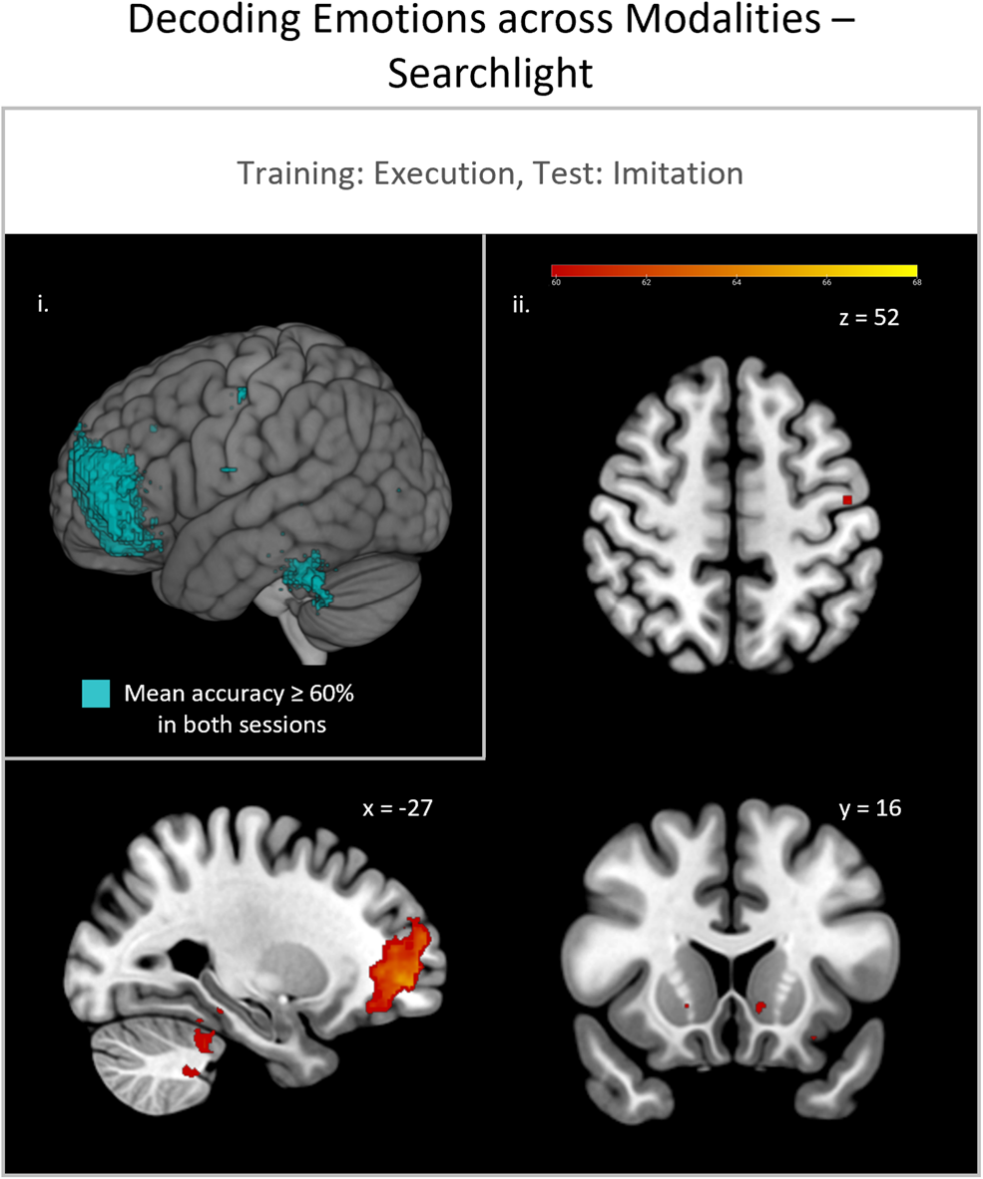
Searchlight analysis for the cross-modal classification of emotions, trained on execution and tested on imitation with cross-session replication. i. Cross-session replication with overlapping significant voxels of study 1 session 1 (S1.1) and session 2 (S1.2) with mean accuracy ≥ 60% binarized. ii. Mean classification accuracy of significant voxels (cut-off: mean accuracy ≥ 60%) of study 1 session 1 (S1.1) only.

In the searchlight analyses, replicable effects across sessions were found when classification of emotional content was trained on the execution condition and tested on the imitation condition within the prefrontal cortex, fusiform gyrus, precentral gyrus, and cerebellum (Fig. 6). The remaining analyses (i.e., training on observation and testing in imitation or testing in execution or training on execution and testing in observation) did not show any replicably significant clusters above threshold.

## 4 Discussion

We assessed the MN properties action specificity and cross-modality within a social-cognitive imitation task using cross-modal MVPA. In addition, we demonstrated cross-session and cross-sample replicability of our findings. Replicable cross-modal classification of emotional content was possible in regions of the MN system and the EFP system when trained on the execution condition and tested on the imitation condition, but not when trained on the observation condition. The searchlight analyses revealed further regions with action-specific and cross-modal representations for execution and imitation mainly in prefrontal areas. To our knowledge, this is the first study to assess the involvement of the MN system in emotion processing using an ROI- and searchlight-based MVPA approach, including replication of the findings in multiple sessions and samples.

As a first step, we classified the social-cognitive conditions pairwise, irrespective of emotional content, and found high accuracies in visual and motor cortex (seeSupplementary Materials). To test our hypotheses, we then first tested for action specificity (in the case of our study, the distinct representation of anger and fear) by classifying emotional content within the single modalities. Neural representations of fear and anger were differentiable when expressions were executed or imitated but not when participants solely observed a picture of an angry or fearful face. This effect was replicated across sessions and across samples. We likewise observed fewer significant regions of the MN and EFP system in a univariate analysis of the data in the observation condition compared to the execution or imitation condition (Schmidt et al., 2021). Although a substantial body of research investigated neural representations of observed emotions, to our knowledge a few studies reported differences between these two emotions with negative valence directly. A study byLiang et al. (2019)reported significant classification accuracies for the differentiation of fear versus anger. In contrast to the present project, film clips were used as stimuli and functional connectivity patterns served as data input for MVPA, which was performed across subjects. More often however, a multi-class or “one-versus-all” approach involving positive and negative emotions is applied to test for information containing regions in general (e.g.,Greening et al., 2018;Harry et al., 2013;Liang et al., 2017;H. Zhang et al., 2016). These types of analyses consider multiple classes (of emotions) in one analysis and therefore do not provide information about differential activation between pairs of classes, in contrast to the binary approach applied in this project.Volynets et al. (2020)observed generally lower classification accuracies for observed than executed emotions. Thus, decoding of observed emotions appears to be more difficult. Therefore, we assume that neural representations of observed fear and anger are similar and difficult to differentiate, as has also been demonstrated for self-induced emotional states of these emotions (Kassam et al., 2013). Neural representations of positive and negative emotions are more dissimilar and might thus be easier to classify. This interpretation is in line with a previous study, in which we identified differential activation in the MN and EFP system to observation of fearful versus happy stimuli with an fMRI adaptation design (Schmidt et al., 2020). One possibility is that our brain’s mirroring mechanism differentiates valence, but not emotions. Future studies could use happy, fearful, and angry stimuli in an adaptation design, to test this assumption. In addition, observation of an emotional expression in others likely not only induces a mirroring mechanism but also an emotional and motivational reaction. Viewing an angry facial expression could pose a potential threat, therefore eliciting a fight-or-flight response in the observing individual with accompanying feelings of anger or fear. This might provide another possible explanation for the lack of findings in the observation condition, as well as lower classification accuracies in the imitation than the execution condition. Moreover, the design of our studies included further demanding tasks during scanning and we did not include “catch trials” during the observation condition. Thus, we cannot rule out that participants were less involved in the task during the observation condition. Differences in motivation and involvement have been found to modulate regional brain activation, also in MN regions (Cheng et al., 2007;Cui et al., 2015). In agreement, a recent study showed higher involvement of MN regions in a grasping / observing interaction in infants than when grasping and observing were isolated and not part of an interaction (Meyer et al., 2022). Thus, activation in MN regions seems to occur particularly reliably when it has an intentional context. Moreover, MN activation during observation has been shown to be higher when facial mimicry is possible (Birch-Hurst et al., 2022). It might be that participants in our study tried suppressing their facial movement in the observation condition, because the other conditions involved active facial movement. To summarize, we found action-specific representations in the execution and the imitation condition, but not in the observation condition. The lack of specific representation in the observation condition might oppose current theories of the MN system by suggesting that pure observation does not activate a motor, let alone a mirroring process. It might also be related to the task context of the observation condition and suggests modifying factors on activation in MN regions when no own facial action occurs.

Secondly, we employed cross-modal classification to test for modality-invariant representations of emotions. As expected, based on non-differentiable representations within the observation condition, we replicated significant cross-modal classification accuracies across sessions only when trained on execution and tested on imitation trials. Thus, the condition of cross-modality for MN properties is only met for these modalities. In addition to*a priori*defined ROIs of the MN and EFP system, the searchlight analysis revealed further information-containing areas with replicable effects in prefrontal, parietal, and occipital lobe, and the cerebellum (Fig. 6). Middle and superior frontal regions have been reported to be differentially activated in the processing of anger and fear (Jehna et al., 2011;Kesler-West et al., 2001). Further, part of the orbitofrontal cortex (Brodmann area 47) has been found to be involved in the recognition of emotions and other social-cognitive processes (Adolphs, 2002;Goodkind et al., 2012;Jonker et al., 2015;Willis et al., 2014). More specifically, it seems to be involved in processing of fearful and angry facial expressions (Palm et al., 2011;Sprengelmeyer et al., 1998). A different explanation for the high classification accuracies in the prefrontal cortex might be the involvement of higher order cognitive processes. When assessing the differential neural response of intention identification,Thompson et al. (2022)found activation in mentalizing regions, including middle frontal and orbitofrontal areas, rather than mirror neuron regions. Moreover, performing or imitating an emotional expression necessarily involves cognitive control processes. On a different note, one might speculate that the large significant prefrontal cluster stems from forehead movement performed when displaying fear or anger expressions, respectively. However, when classifying the experimental conditions (regardless of emotional content), largest classification accuracies were not found in prefrontal areas but in motor and visual cortices. Moreover, significant areas in the searchlight analyses lay mainly in grey matter regions, indicating that there is no general movement bias. Additionally, we performed control analyses probing the robustness of our findings and exploring the influence of movement on the classification accuracies (seeSupplementary Materials). We demonstrated that emotional content could neither be classified by movement parameters alone nor that the amount of movement during execution and imitation trials would facilitate decoding. These findings show that participants’ movement did not significantly influence classification accuracies. However, it is still possible that the exact muscle movements influence classification.

Albeit MVPA is a well-suited candidate to assess MN properties in humans (Oosterhof et al., 2013) with high sensitivity up to sub-voxel scales (Kamitani & Tong, 2005), results remain inconsistent across studies. Even in monkeys, where single-cell recordings provided convincing evidence for the existence of MN, cross-modal fMRI assessments do not consistently show cross-modal and action specific representations (Cui & Nelissen, 2021;Fiave et al., 2018). It thus seems even more crucial to assess whether findings are replicable. Although there are studies reporting high reliability of MVPA results in general, to our knowledge only a few studies assessed this quality criterion (Kragel et al., 2021;Taxali et al., 2021). This is especially true for within-subject classification designs (Han et al., 2022). For the results of the presented project, we could not only assess replication over repeated scanning sessions but for part of the analyses also over different samples. Our interpretations are thus based on results that we found to be replicable. Consequently, our reports are stricter than in previous studies, for example, significant cross-modal classification was found in regions of the EFP system within one session, but not the other (Fig. 5).

At least five limitations of the presented study should be considered and may inspire future studies. 1) The tasks used in our studies were not specifically designed for MVPA analyses, and the primary aim was not the differentiation of emotional content, but to investigate the MN system in social cognition. Thus, only fear and anger could be investigated for the present analyses, and we had fewer trials per modality and emotion than in other cross-modal MVPA studies. Also, we acquired only one run per participant. We thus only interpreted replicable results. With an adaptation design, we could recently demonstrate that the regions of the MN system differentiate between valences, opening the possibility that studies with more trials and more distinct facial emotions would find cross-modal and action specific representations also when the observation condition is included (Schmidt et al., 2020). 2) Due to a restricted size of the FOV or increased levels of noise, we observed signal loss in frontopolar, dorsal parietal, anterior temporal, and cerebellar regions, making it impossible to explore within-subject classification in these regions. 3) Experimental set-up and scanning parameters differed between samples. In S1.1, simultaneous EEG-fMRI measurements were performed. It was reported that EEG measurements do not substantially influence reliability of fMRI data (Klein et al., 2015;Luo & Glover, 2012). In addition, we have no reason to believe that the task conditions would have been systematically influenced differently. However, we cannot rule out such influences. In S1.2, participants included in the analyses received sham TMS immediately before scanning. Since they did not know whether they had received sham or real TMS, a placebo effect might have occurred that influenced their perception of and reaction to the stimuli, thereby possibly reducing the replicability of results. However, these influences would impede replication between our sessions and samples and thus rather point towards a robustness of our findings. 4) Our choice of classification parameters was informed by standards in the field and recommendations based on the utilized TDT toolbox (Hebart et al., 2014). However, different classifiers, such as random forests and gradient boosting, or feature selection methods could further augment classification performance (Douglas et al., 2011;C. Zhang et al., 2017). 5) To the best of our knowledge, a “gold standard” for significance testing in within-subject classification designs has yet to be established. Although the applied two-step permutation scheme has been criticized (Allefeld et al., 2016), it is an approach often utilized and alternative approaches have been found to lack power (Hirose, 2021).

In conclusion, while we reveal high classification accuracies that are replicable across different sessions and samples, our project does not fully replicate MVPA findings of shared neural representations for observed and executed facial emotional expressions (Volynets et al., 2020) or other movements (Etzel et al., 2008;Oosterhof et al., 2010,2012a,2012b). However, for execution and imitation of emotional facial expressions, we could demonstrate that regions of the MN and EFP system, as well as further regions including prefrontal areas, exhibit action specificity and cross-modality of representations. Since imitation constitutes a compound of perceiving and performing, and thus both imitation and execution include active displaying of the emotions, our results only partially lend support to the embodied simulation theory. The lack of findings for the observation condition contradicts the idea of a common neural representation for perception and action. However, this might emerge from the similarity of representations of observed negative emotional expressions or from diminished motivation and involvement due to our task design. As proposed in the recent literature, assessing reciprocity and synchronization in direct interactions might be a promising avenue, as it not only ensures task engagement and ecological validity, but also allows to investigate prediction of behaviors (Bonini et al., 2022;de Gelder, 2023). Furthermore, a differentiation of action identification and intention identification to study MN showed a specific involvement of different brain regions in these processes (Thompson et al., 2019,^2022^). Future studies should investigate cross-modality and action specificity by applying tasks with clear differences in emotional valence of the facial expressions and by ensuring consistent task engagement to further explore mirror properties of the brain in social cognition.

## Supplementary Material

Supplementary Material

## Data Availability

Researchers who are interested in getting access to the data that support the findings of this study should contact the author Stephanie Schmidt (Stephanie.3.Schmidt@uni-konstanz.de). They will be granted access to anonymized data to use it personally, but are not permitted to distribute it further. The conditions of our ethics approval do not permit public archiving of our MRI data. Group result maps are available on OSF:https://doi.org/10.17605/OSF.IO/3CXYR. Code used in the presented project is publicly available from the TDT toolbox (Hebart et al., 2014).
