# Supplementary material for: Cross-modal decoding of emotional expressions in fMRI—Cross-session and cross-sample replication
Source: Imaging Neurosci (Camb). 2024 Sep 23;2:imag-2-00289. doi: 10.1162/imag_a_00289 (PMC12290836; doi:10.1162/imag_a_00289)
Supplement: Supplementary Material [file imag_a_00289-supp.pdf]

Supplementary Material

Decoding modalities

To classify modalities, all beta images of the conditions irrespective of the emotional content were used as classifier input, respectively. Analyses were performed equivalently to the describe procedures including a 5-fold cross validation scheme, the two-step permutation approach and assessment of replication. Only for the classification of imitation and control solely data of study 2 (S2) were used due to unbalanced classes in study 1.

For the classification of observation versus execution and imitation versus control, highest classification accuracies were found in visual and motor cortices (Fig. S1 A & D). Accuracies were highest in motor cortex for the classification of imitation versus observation (Fig. S1 B) and in visual cortex for imitation versus execution (Fig. S1 C).

Decoding Modalities – Searchlight

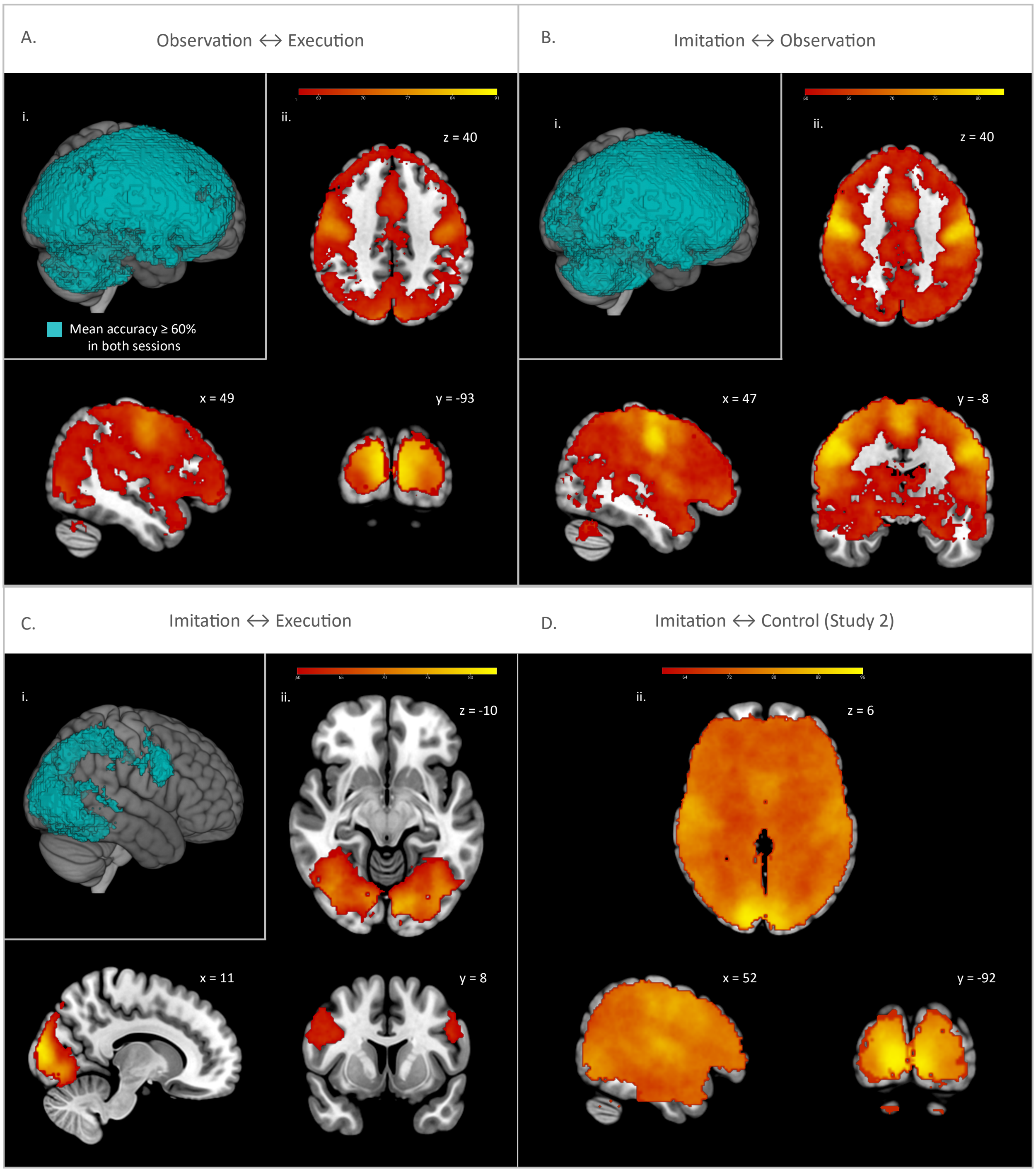

Fig. S1. Searchlight analyses for the classification of modalities. (A) – (C) show mean accuracies for classification of modalities in study 1. (D) shows mean accuracies for the classification of the imitation and control condition in study 2 (S2). i. Cross-session replication with overlapping significant voxels of study 1 session 1 (S1.1) and session 2 (S1.2) with mean accuracy ≥ 60% binarized. ii. Mean classification accuracy of significant voxels (cut-off: mean accuracy ≥ 60%). For (A) – (C) only results of study 1 session 1 (S1.1) are depicted, for (D) only of study 2 (S2).

Control analyses

Our main results showed that classification of fear and anger was only possible within and across the execution and imitation condition, but not the observation condition. To explore a possible influence of differential facial movements of these two emotions on decoding ability, we performed two control analyses. First, we tested whether fear and anger could be classified from movement parameters alone, in data from study 1 session 1 (S1.1). For this purpose, we classified emotional content within the execution and imitation condition respectively, with only movement parameters from realignment during preprocessing as classifier input. To this end, for each possible movement (3 translations, 3 rotations) we calculated the mean of the difference in movement between the scan in which the trial began and the preceding one, as well as between the scan in which the trial began and the following one. Since participants were instructed to display the expression for the whole trial, the initiation and with this the highest amount of facial movement occurred at the beginning of the trial. Classification settings were kept the same as described for within-modality classifications, including the 5-fold CV scheme. We found a mean classification accuracy of $M = 51.92$ ($SD = 10.92$) for the execution condition and $M = 52.33$ ($SD = 10.87$) for imitation. These values are just above chance level and lie far below accuracy results within ROIs (Fig. 2) and whole-brain searchlight results (Fig. 3 & Fig. 4). Moreover, classification accuracies based on movement parameters did not correlate with accuracies in the MN and EFP system, neither in the execution nor the imitation condition.

Second, we assessed whether there is an association between the amount of movement and classifier performance (see Fig. 3). For this, we calculated the mean classification accuracy over voxels lying within the frontal clusters found in the searchlight analysis for overlap in the execution and imitation condition separately and per participant of S1.1. As a proxy for movement quantity, the mean framewise displacement (FD) from the first two volumes of each trial was calculated according to Power et al. (2012). The correlation between mean accuracy in the frontal cluster and mean FD for execution was $r = -.009$, $p = .937$ and for imitation $r = .127$, $p = .287$. Additionally, we assessed the correlation between ROI results and movement quantity. In the execution condition, mean FD did not correlate significantly with classification accuracy in the MN or EFP system. In the imitation condition, there was a significant negative correlation between mean FD and classification accuracy in the EFP system $r = -.371$, $p = .001$, but not in the MN system. Hence, the larger the movement at the beginning of imitation trials, the lower was the classification accuracy based

on neural activation patterns in the EFP system. Therefore, movement rather seemed to impede than improve classification of fear and anger.

References

Power, J. D., Barnes, K. A., Snyder, A. Z., Schlaggar, B. L., & Petersen, S. E. (2012). Spurious but systematic correlations in functional connectivity MRI networks arise from subject motion. Neuroimage, 59(3), 2142-2154. https://doi.org/10.1016/j.neuroimage.2011.10.018
